# Colonisation and mass rearing: learning from others

**DOI:** 10.1186/1475-2875-8-S2-S4

**Published:** 2009-11-16

**Authors:** Mark Q Benedict, Bart GJ Knols, Hervé C Bossin, Paul I Howell, Eric Mialhe, Carlos Caceres, Alan S Robinson

**Affiliations:** 1 Entomology Unit, FAO/IAEA Agriculture and Biotechnology Laboratory, IAEA Laboratories, A-2444 Seibersdorf, Austria; 2 Div. Infectious Diseases, Tropical Medicine & AIDS, Academic Medical Center, F4-217, Meibergdreef 9, 1105 AZ Amsterdam, The Netherlands and K&S Consulting, Kalkestraat 20, 6669 CP Dodewaard, The Netherlands; 3 Institut Louis Malardé, BP 30, 98713 Papeete, Tahiti - Polynésie Française; 4 Centers for Disease Control and Prevention and Atlanta Research and Education Foundation, 4770 Buford Hwy, Atlanta, GA 30341, USA; 5 Concepto azul S.A. and Univ. Guayaquil, Guayaquil, Ecuador; 6 USDA - APHIS, 12 Av. 4-65 Zona 10, Guatemala City, Guatemala, 01010

## Abstract

Mosquitoes, just as other insects produced for the sterile insect technique (SIT), are subjected to several unnatural processes including laboratory colonisation and large-scale factory production. After these processes, sterile male mosquitoes must perform the natural task of locating and mating with wild females. Therefore, the colonisation and production processes must preserve characters necessary for these functions. Fortunately, in contrast to natural selection which favours a suite of characteristics that improve overall fitness, colonisation and production practices for SIT strive to maximize only the few qualities that are necessary to effectively control populations.

However, there is considerable uncertainty about some of the appropriate characteristics due to the lack of data. Development of biological products for other applications suggest that it is possible to identify and modify competitiveness characteristics in order to produce competitive mass produced sterile mosquitoes. This goal has been pursued - and sometimes achieved - by mosquito colonisation, production, and studies that have linked these characteristics to field performance. Parallels are drawn to studies in other insect SIT programmes and aquaculture which serve as vital technical reference points for mass-production of mosquitoes, most of whose development occurs - and characteristics of which are determined - in an aquatic environment. Poorly understood areas that require further study are numerous: diet, mass handling and genetic and physiological factors that influence mating competitiveness. Compromises in such traits due to demands to increase numbers or reduce costs, should be carefully considered in light of the desired field performance.

## Background

Making "a better mosquito" that is suitable for the sterile insect technique (SIT) requires producing and releasing sterile males in large numbers, which then compete successfully against wild males for virgin wild females. Therefore, colonisation and production methods that lead to such an idealized mosquito must be developed. In this restricted sense of what defines a better mosquito, artificial programmes, similar to animal breeding for desired qualities, can theoretically attain a higher or at least equal level of performance than evolutionary selection. The multitude of traits refined by natural selection is necessarily reduced in number for the purposes of SIT, and very low "fitness" mosquitoes may be suitable for SIT. Indeed because fitness is measured by reproductive success, the fitness of sterile males is zero - by design.

Rather than attempting to produce 'natural' mosquitoes, attempts will be made to cultivate mosquitoes in ways similar to the improvement of specific traits of agricultural plant and animal commodities that are accomplished by breeding and cultivation methods. Traits such as per hectare yield, sugar, protein, and fat content and disease resistance have been successfully modified. Given mosquitoes' adaptability to the laboratory, short generation time, ability to develop on many different diets, and measurable performance characters, it is expected that traits relevant to SIT can be deliberately improved or, at least, maintained. As the reader will see though, there is considerable uncertainty about exactly which measurable characters should be the focus.

## Colonisation of mosquitoes

### Evolution and genetics of colonised mosquitoes

Of all the life-history traits that present difficulty in the transition to the laboratory, mating is the most problematic [1] but is essential to any attempt to establish a colony. This character is at the heart of successful SIT operations as well, yet little is known about specific environmental conditions that promote it [2].

Although most major *Anopheles *malaria vector species have been colonised for laboratory rearing (see additional file 1), for SIT applications it is logical to ask, "Would these males mate with wild females and at what rate relative to wild males?" The quantitative measure of this trait is termed competitiveness [3]. Population bottlenecks, genetic drift, deliberate and inadvertent selection, rearing, sterilisation and release methods can all reduce competitiveness. It is generally appreciated that hybrid vigour contributes to improvement of traits of many organisms, and it is possible that crossing and colony maintenance to achieve similar effects in mosquitoes would be useful. Adult size, and the related characters fecundity and wing length, were significantly increased by hybridizing two strains of *Anopheles gambiae *thus providing promising support for this approach [4]. It will now be important to determine whether these observations translate into increased mating competitiveness. While it is widely assumed that maintaining natural genotypes or increasing heterozygosity *per se *are desirable to obtain competitive males, a literature review identified no experiments with mosquitoes in which this has been explicitly tested. Laboratory experiments in which heterozygosity was incidentally increased did not demonstrate an increase in competitiveness as a result [5].

Differences between mass-produced and natural mosquitoes begin to accumulate during colonisation. Such effects of colonisation and inbreeding on the genetic composition of the colony are commonly viewed from a probabilistic standpoint using founder, drift and selection models. From this perspective, colonies become increasingly homogeneous entities that are genetically very different from wild populations and whose competitiveness is assumed to decline. While this view describes general trends [6], there are countering forces whose genetic bases are poorly understood, particularly the role of lethals and natural recombination suppressors (discussed previously [7]). As an example of such effects, two inbred strains of *Aedes triseriatus *undergoing full-sib mating for at least 12 generations were analysed for isozyme polymorphism [8]. In spite of intense inbreeding, a significantly higher-than-expected level of polymorphism was observed at several loci. The authors concluded that *heterozygosity itself *is a strongly selected character in inbred populations and that the mechanism in this case was the presence of recessive lethals resulting in balancer systems. While the effort to accomplish genetic homogenisation that was conducted in other studies [9] was not as intense, heterozygosity was maintained at a level near, or higher than, the parental material, possibly again reflecting selection for heterozygosity.

A similar natural phenomenon has been observed in anophelines, including members of the *Anopheles gambiae *complex. Unexpectedly high levels of paracentric inversion polymorphism have been observed in field and laboratory populations of *Anopheles gambiae s.s*. [10]. While this may be co-selected by insecticide resistance alleles linked to the inversions, heterosis independent of such selection is likely. A heterozygous chromosomal rearrangement present in excess of the expected frequency in field and laboratory populations of North American *Anopheles quadrimaculatus *[11] again suggests that natural balancers may be common. Until the genetic mechanisms and frequency of such balancing systems is determined, a cautious approach to anopheline colonisation will be to maintain and increase polymorphism insofar as is practical. In order to provide experiment-based direction on this subject, a useful research initiative would be to conduct experiments that would specifically address this question. This information would be valuable for those implementing SIT and other genetic control projects.

Concerns about behavioural and genetic incompatibility have resulted in a default approach to select a release strain for colonisation that originated in the target population locale. While entirely defensible, this approach ignores possible beneficial effects of heterosis that might be obtained by producing geographic hybrids and choosing strains with intrinsically superior competitiveness. Several factors of importance to production and mating competitiveness have a genetic component: longevity and fecundity particularly appear to be amenable to manipulation by strain selection and crossing [12]. Until laboratory surrogate indicators that accurately predict field performance are devised, it will be necessary to conduct colonisation and competition experiments on a more ambitious scale than has been performed previously.

### Promoting mating during colonisation

Mosquitoes clearly mate satisfactorily in the field, but simulating this in the laboratory is difficult, presumably due to differences between the environment of the laboratory and natural one. Strenuous efforts to simulate natural lighting, temperature, humidity, presence of other swarming mosquitoes, and spaces have been made, but it is difficult to conclude how effective many measures are. Marchand concluded that an artificial horizon stimulated swarming behaviour of *An. gambiae *and *Anopheles arabiensis*, but that ground markers in the cage had no noticeable effect [13]. Gradual light changes to simulate sunset and sunrise are also useful [2], however, knowledge of which of the multitude of factors being simulated is necessary is often lacking during colonisation and stock-keeping, and it is even less clear as to whether they ultimately affect the performance of the mosquitoes that are colonised. Comparison of semi-natural vs. laboratory colonisation of *Culex tarsalis *was performed in either a constant or variable environment beginning with identical field-derived cohorts [9]. In an attempt to simulate a more natural environment, variation in temperature, oviposition containers, cage size, and resting sites was provided to one group while constant conditions were offered to the other. The experimental question was, "Which condition resulted in mosquitoes that were more similar to wild type after 12 generations?" Indicators included fecundity, development rate, size, mating competitiveness and genetic similarity to the founder material. While the authors concluded that the variable environment resulted in a colony genetically more similar to the wild type, both had similar high levels of heterozygosity, and laboratory adaptation still occurred, albeit at a slower rate in the natural simulation. Until experiments identify specific environmental factors that minimize directional selection, mosquito SIT programmes must proceed without clear guidance of which environmental factors must be manipulated to avoid it.

## Classical genetic sex separation strains

### Genetic considerations of traditional genetic sex separation strains

A mosquito species' propensity to mate in confined (i.e. laboratory cages) vs. open spaces is termed stenogamy vs. eurygamy. Preliminary evidence from *Anopheles maculipennis *complex mosquitoes suggests that this propensity is controlled by the Y chromosome [14], but similar studies to confirm this have not been performed in other *Anopheles*. If the following two propositions are true, (1) the Y chromosome does in fact control stenogamy/eurygamy, and (2) these characteristics are important for mating competitiveness; then it is critical that genetic sexing strains (GSS) be created using a Y chromosome that carries traits necessary for mating with the target population. Because traditional anopheline sexing strains have been created by linking a dominant marker to the Y chromosome, introgression of Y chromosomes - and stenogamy/eurygamy - from a wild target population into such a sex-separation strain will be impossible because there is no opportunity for recombination between Y chromosomes. Encouraging, but limited, experience in El Salvador with the *Anopheles albimanus *MACHO sex separation strain confirmed field competitiveness, even though no preliminary tests assured competitiveness of the stock from which it was created [15]. Classical sex-separation strains have disadvantages of reduced fertility and breakdown due to recombination, but they are still useful because they are not subject to special regulations imposed upon transgenic insects. The disadvantages listed above are characteristics over which transgenic mosquitoes might have advantages [16,17]. For example, they would not have reduced fertility, and they would allow introgression of wild genotypes to a greater extent than a classical strain in which a large proportion of the chromosomes experience reduced recombination due to rearrangements.

Beside competitiveness considerations, the introduction or modification of genotypes may be required for introducing specific characters into laboratory and production colonies. Outbreeding is the most apparent solution. This strategy rapidly introduced wild genotypes into *Culex nigripalpis *[18], however, it required the use of field-collected males crossed to colonised females. Because wild males will mate colonised females more readily than wild females will mate colonised males [2], this strategy cannot be implemented in a single generation as it is inimical to introgression of wild material into classical Y-translocation sexing strains in *Anopheles*.

Alternatively, however, an intermediate strain produced by repeated crossing of field-collected males to colony females could provide suitable progeny females to which males from the sexing strain could be mated. Repeated introduction of wild material was employed to create a vigorous wild-type *An. albimanus *strain, CAMPO, but without genetic assessment during the El Salvador SIT releases [15].

### Maintaining genetic sexing strain integrity

A single GSS selection genotype has been used for the control of Mediterranean fruit flies *Ceratitis capitata *globally in area-wide SIT programmes releasing four billion sterile males per week [19]. This is based on the use of classic genetic approaches in which a male-linked chromosomal translocation links wild-type alleles of a pupal colour mutation and normal temperature sensitivity to the male sex chromosome. Females carry a white pupa mutation and are temperature sensitive.

Like the low frequency of recombination between the insecticide resistance marker and the Y chromosome seen in the MACHO strain of *An. albimanus *(which results in resistant females), *C. capitata *GSS are not absolutely stable and require elimination of unwanted recombinants while not interfering with production. The filter rearing system (FRS) is a management strategy that was designed to remove the products of genetic recombination and hence maintain the integrity of the GSS [20]. The FRS is based on the use of a relatively small colony from which recombinants are removed and from which eggs are collected. Through a process of amplification for several generations, sufficient male insects are produced for sterilisation and release. Depending on the stability (i.e. frequency of recombination events), the FRS colony should be purified each generation, and it must be large enough to produce sufficient offspring to support itself and to initiate the colony amplification. Because the FRS is a unidirectional system of production, it also provides a generic system to control additional quality aspects. For example, a new small colony that preserves sexing characteristics refreshed with wild genotypes can be introduced on a regular basis to replace the broodstock to prevent accumulation of "lab-adapted genotypes" or to otherwise modify the genetic composition of the colony. The FRS can also be maintained under less stressful conditions in order to maintain important quality traits.

## Mass-rearing

It is no surprise to people rearing mosquitoes that the basic utensils and methods of mosquito rearing are similar to those described 60 years ago [21]. Personal experience and inherited methods rather than controlled experiments have been the mainstay for developing mosquito rearing, and these methods are sufficient for laboratory use. In the following section, some of the practices are discussed that have been helpful to large-scale production efforts. Simple innovations are also suggested with potential to simplify and improve mass rearing.

### Management

Effective management systems are absolutely critical for the success of production and distribution of millions of sterile mosquitoes. The statement [22] that, "...the failure of area-wide programmes is usually due to poor management and inadequate public support and not to poor technology." is a widely recognized truth. Good management requires methods similar to those used in business and industrial mass-production - disciplines in which few scientists who are charged with this responsibility have any training. Notably, the two largest release programmes quickly adopted such management systems and carefully controlled production levels and quality analysis. Most notably the World Health Organization/Indian Council of Medical Research (WHO/ICMR) programme attributed its successes to reliance on the Program Evaluation and Review Technique (PERT) [23]. This method is still widely used and provides a useful model for programme management. The production of *An. albimanus *in El Salvador required careful planning and analysis of production systems which have been well described [24]. However, no overall management system of that project has been described. Of great value to wider application of mosquito SIT will be development of a standard model of management system for the operation of facilities worldwide.

### Production systems

Because previous mass-rearing efforts have essentially increased the scale of laboratory methods, integrated production systems are an area in which rapid dramatic improvements can be expected. Several infrastructure improvements could significantly improve the quality of rearing: circulating water systems with continuous quality monitoring and waste removal, continuous larval/pupal separation and sterilisation systems, measures to reduce and prevent the development of pathogens, and feeding systems that maintain optimal diet availability.

In spite of the demands and scale of mosquito release programmes, it is surprising that the infrastructure differed mainly in number from that used in laboratories for small scale rearing. While the WHO/ICMR used a centralized aquatic stage collection system, the El Salvador *An. albimanus *SIT project relied on individual rack-mounted trays. Both programmes utilized pupal separation methods and adult cages nearly identical to those used in research laboratories. Neither mechanized nor continuous methods for blood feeding, egg collection, larval feeding or pupal separation were implemented. Useful models exist however: on a small scale, a circulating system that included biological water conditioning and automatic feeding for rearing of *Anopheles stephensi *provides an example of how such systems can be created [25].

The economic considerations that limited the development of mechanized specialized systems decades ago are now a smaller obstacle because of the availability of inexpensive personal computers, software, control devices, and solid-state sensors. In the final analysis of the utility of SIT, economics of production will make developing these systems essential. The low cost of labour in disease endemic countries should not be used to justify a lack of technology development. Investing in the training and infrastructure of the persons working in the factory and the host country will be better served by transferring efficient advanced technical skills rather than instruction in menial labour. Furthermore, strikes, absenteeism, and inconsistent performance are less likely to interfere with a production system that does not depend excessively on numerous personnel. Finally, some programmes have found it difficult to decrease staff sizes when increased efficiencies and demand resulted in diminished labour needs. Starting with as small a staff as possible reduces these problems.

### Larvae and pupae

Larval rearing conditions have a direct, and often irreversible, effect on adult traits therefore a clearly defined diet is a priority. Developing such a diet is not as straightforward as it might appear and is usually accomplished by trial and error. Because mass rearing has not been performed under aseptic conditions and mosquito larvae are opportunistic feeders, microbes constitute a large portion of their diet, and no truly defined diet for mosquitoes has been used in a large-scale system. Regardless of the fact that controlled conditions are desirable for mass production, and aseptic defined-diet rearing of mosquitoes would help accomplish this, it will likely not be practical on a large scale.

A chemically defined diet was used to successfully rear several culicines [26], but was not successful for *Anopheles freeborni *[27], though small-scale sterile anopheline rearing is possible [28]. In an interesting example of the hunter becoming the hunted, Munderloh *et al*. [29] were able to rear *An. stephensi *larvae on cultured cells of a mosquito that itself preys on mosquito larvae, *Toxorhynchites*. An ideal diet would provide adequate nutritional components that positively influence rearing conditions and insect quality, is inexpensive, globally available, and of consistent quality. Those that are used for smaller scale culture such as TetraMin™ Flake Food are of high quality but of high cost and would not be desirable for mass-rearing. As an indicator of the amount of diet required - and the significance of cost - we have estimated that approximately 2-3 kg of larval diet per day will be required for production of one million males per day. Mass rearing diets have consisted of inexpensive ground dried animal foods (sometimes defatted to prevent 'scumming'), cereals, yeast, beef liver powder - and of course the happenstance unidentified microorganisms. Conversely, cat and monkey chow are often used, but are neither designed from mosquito larvae nor can they be obtained in the same form globally. Successful combinations were determined empirically, but when used under septic conditions, it is impossible to say to what extent the diets were useful because either they fed the larvae directly, supported the microorganisms on which the larvae fed or both. In response to these concerns, the IAEA mosquito SIT programme is currently testing various ingredients that we feel meet the necessary requirements described above e.g. beef liver powder, yeast, fish meal and squid liver meal (unpublished data).

#### Key issues for colonisation and production

• *What genetic factors control eurygamy/stenogamy?*

• *What is the relationship between stenogamy and field mating competitiveness?*

• *What are the genetic and mating consequences on colonised mosquitoes of cage introductions of field-collected individuals, and what method best accomplishes genetic introgression of wild genotypes into colonies?*

• *Does genetic heterogeneity affect laboratory vigour and mating competitiveness?*

• *What is the relationship of dispersal capacity - as measured in the laboratory using behavioural and biochemical tests - to actual field dispersal behaviour?*

• *Do tactile interactions of mosquito larvae affect development independently of related factors such as food availability and waste accumulation?*

• *Do various geographic strains of a species have different levels of competitiveness for females of a target population?*

• *Can artificial selection increase male competitiveness? If so, what characters are altered in the process?*

• *What is the relative importance of dispersal behaviour to program effectiveness under various population density and distribution scenarios?*

• *What do male age, size, and longevity contribute to mating competitiveness?*

• *Do specific dietary components not reflected in gross energy reserves affect competitiveness?*

Experimental guidance exists regarding dietary components that affect competitiveness. A rare, clear example exists where a specific dietary component was elegantly linked to field performance of mosquitoes and has influenced our choice of test diets described above. During a series of release experiments in California, a dramatic reduction in first generation *Cx. tarsalis *mating competitiveness was observed [30]. Because these males were genetically identical to field males, laboratory rearing factors were suspected. (*n.b*.: This should be considered as an excellent routine method to determine the effect of factory production alone on competitiveness independently of genetic effects.) A marked reduction in fatty acids in laboratory reared mosquitoes had previously been observed relative to wild type [31] and linked to flight ability [32]. A series of experiments identified several fatty acids that were reduced in laboratory mosquitoes reared on a defined diet and that were necessary for flight and competitive mating. As a result, the authors recommended supplementing the diet with fish oil or other sources of eicosapentanoic acid, a consideration not heeded by many laboratories that have carefully avoided fatty diets for mosquito larvae. More recent experiments conducted by Huho *et al*. [33] demonstrated that total lipids were less abundant in laboratory reared mosquitoes relative to wild type though no attempt was made to analyse specific fatty acids. Both of these series of observations should focus attention on the importance of controlling the supply of fatty acids in the factory independently of total weight, lipid content and growth rates.

Spatial and tactile interactions in the larval environment are also poorly understood, due largely to the lack of appropriate experimental equipment that isolates the effects of larval and diet density, total diet amount and availability and waste accumulation. Timmermann and Briegel [34] clearly demonstrated that increasing water depth adversely affects anophelines but not *Aedes*, and inhibitory tactile interactions have been detected in *Culex sitiens *[35], but the absence of circulating/exchanging water systems and a constant diet concentration and amount makes it difficult to separate interacting variables.

A practical compromise between an undefined and defined aseptic rearing system is possible and has not received significant attention: numerous species of microorganisms (e.g. *Tetrahymena, Euglena, Chlamydomonas*) can be produced in large amounts, are of an appropriate size, and would provide a complex food for larvae. Advantages of many such protists are that they scavenge unwanted bacteria and fungi and can be aseptically cultured in simple medium in large amounts. Moreover, many of these are motile and accumulate at the water surface where most larval feeding occurs. These might provide a controlled complex diet that would be compatible with sanitation, mass-production, cost requirements, automation, and importantly, anopheline feeding behaviour.

In conclusion, until studies based on clear and relevant adult male mating performance outcomes are completed, a conservative approach is to choose a complex diet containing animal fats, even if it is undefined. As basic as this element is to the success of SIT, diet development is a high priority for efforts to mass-rear mosquitoes, and adequacy of diets cannot be determined merely by one's ability to consistently rear mosquitoes in the laboratory.

### Pupa collection

**Figure 1:**
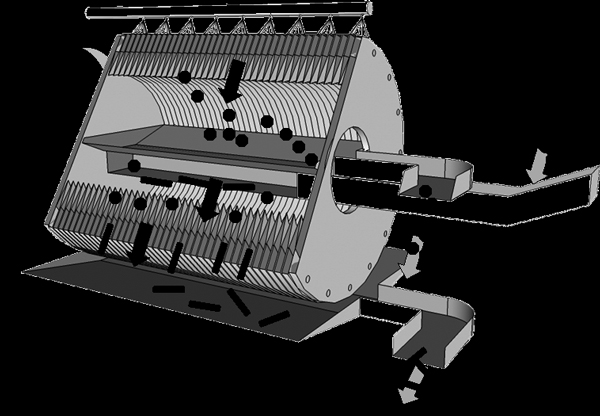
**Cut-away view of a concept pupa-larva separator for continuous mechanized operation**. A continuous mixed stream of larvae and pupae flow into a rotating drum consisting of parallel tapered discs. The anticipated diameter of the drum is approximately 40 cm. Because of their greater effective size, pupae (circles) are captured in the slots whereas larvae (rectangles) flow through and are caught in a tray from which they can be returned to culture trays to complete development. As the drum rotates, the pupae move upward and beneath a spray bar dislodging them into a collection tray. The design could be modified for various-sized species by changing the disc taper and amount of the space between disks.

In some laboratories pupae are not separated from larvae but rather adults are collected directly from larval trays, a method that will likely not be practical for mass rearing. However, unless rearing methods are devised that result in synchronous pupation of a large proportion of larvae in a single day or two, we assume that in mass-rearing systems, pupae will be separated from larvae prior to adult emergence. Fortunately, size, behaviour, and buoyancy differences between larvae and pupae allow selection by means that are readily amenable to mass production and mechanization. To stimulate thinking toward this goal, a conceptual separator is shown that could operate continuously rather than in a batch-wise manner (Figure 1). The basis of the separation is the greater diameter of pupae relative to larvae: since female pupae are larger than males in some species, it might also be suitable for sex separation. This device would have several advantages over previous methods: larvae and pupae could be automatically counted in the stream for redistribution to rearing containers and release, partial or complete water and food changes could be performed automatically, and separations could be performed several times daily from a cohort to provide precisely staged pupae for irradiation.

### Adults

Sucrose, glucose and fructose solutions are common adult diets, though fruits are sometimes provided, such as raisins. The use of anti-oxidants in diets to increase longevity and fecundity e.g. [36,37] should be considered and implemented as a routine measure. One report described a complex vitamin mixture which was offered to mosquitoes during colonisation, but no information was provided about its effectiveness [38]. The simplicity of designing and conducting survival and fecundity experiments to test various supplements should lead to greatly improved adult diets. A common food preservative for human diets, methylparaben, has been shown to increase adult longevity of anophelines [39] and was adopted by that programme for routine use. For mass production though, additional information on fecundity would be needed prior to implementation.

For blood-feeding a large number of female mosquitoes, defibrinated blood or blood treated with an anti-coagulant, is provided via a membrane feeding system. Fecundity is reduced in these systems, and the absence of human pathogens in the blood meal must be assured. An ideal blood meal would be totally synthetic, but continued reliance on treated natural blood should not prevent production of competitive adults. For relatively small amounts (e.g. several litres per week), blood can be collected sterile in veterinary bags from cattle and used fresh or after refrigeration. Larger volumes would be problematic as measures to sterilise the blood after collection in an abattoir might be necessary. If this is required, methods for preparation and storage of bovine blood have been developed and tested for tsetse rearing [40,41]. Commercial companies will supply rather large volumes of aseptically collected animal blood but at a relatively high cost.

**Figure 2:**
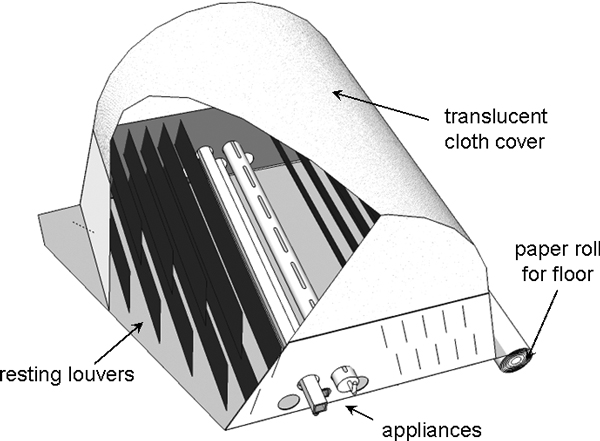
**A concept adult colonisation and production cage**. The cage base measures approximately 1 × 1 m and is approximately 1.8 m high. The bottom sides are rigid and consist of aluminium sheets. The sides and dark resting louvers slope inward to minimize excretion of resting adults onto cage mates. The bottom of the cage is covered with newsprint paper fed into the cage from a roll to allow simple renewal. The appliances consist of devices for egg collection, pupa introduction, blood and sugar feeding tubes. Each is open over part of its length for adult access. The tubular design allows removal of the tubes for cleaning and would allow solutions to circulate through them if desired. All activities associated with such a cage could be mechanized or centrally performed without opening the cage. A cage of this design has been constructed by the authors as have many of the appliances. An artificial horizon as described by Marchand [13] is created between the lower dark portion and the upper translucent cloth cover. We have observed mating swarms occurring at the interface as by Marchand (Figure 3).

**Figure 3:**
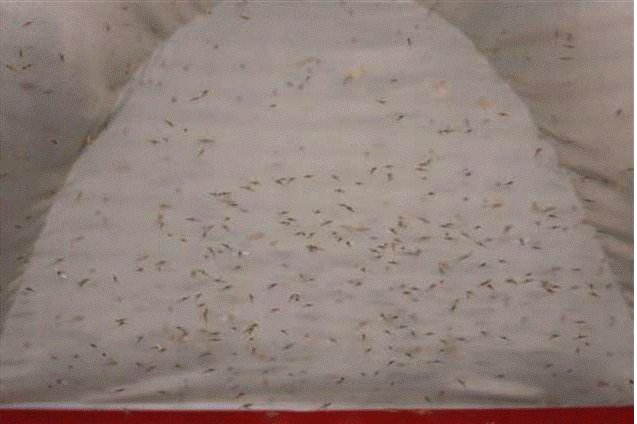
**Adult *An. arabiensis *swarming**. The effectiveness of the artificial horizon in the cage shown in Figure 2 is seen by swarming occurring during an artificial dusk.

Adult rearing usually consists of a larger number and size of cages than those used in numerous research laboratories with little consideration of adult biology. A multitude of concepts should be reconsidered for the next generation of adult rearing cages and tested by experimentation. These are the needs for mating arenas, sanitation, egg collection, blood-feeding, and adult resting behaviour. A concept for such a cage is shown in Figure 2. Several features of such a cage might meet these requirements and be consistent with large-scale production. Similar considerations to those that resulted in this concept cage should be applied to other prototypes that are capable of holding large numbers yet require little maintenance. Preliminary testing and modification of this cage has indeed demonstrated mating at the artificial horizon (Figure 3), but we have found (unpublished data) that extensive modification to the egg collection device is needed. The removable paper bottom also functions well to prevent waste accumulation, and we have retained this feature in a second prototype, which is stocked with 40,000 adults. Like the first version, it retains extensive adult daytime-resting sites, which *An. arabiensis *prefer.

### Controlling disease in the factory

Interventions for disease control have consisted largely of routine hygiene, bleaching trays and equipment, use of disposable items etc. No air and water filtration sufficient to prevent contamination has been used. If selection for greater competitiveness or ease of production in the factory occurs at the expense of immuno-competence, this will become of greater concern.

In previous mosquito release programmes, measures for disease control have been conducted without knowledge of potential pathogens in the colony. Given the paucity of information, past experiences with pathogens in mosquito mass-rearing systems are encouraging. For instance, mass rearing of *An. albimanus *in the early stages in El Salvador was fraught with a high number of "bad trays" (10%-16%) from which few or no pupae were harvested. Often these trays exhibited lack of synchrony in development and foul-smelling water suggesting that improper amounts of diet were provided. Later, when the rearing was increased to one million male pupae per day, losses were reduced to around 1%. In both circumstances, eggs were cleaned simply by washing with water, which apparently sufficiently reduced *Nosema *and/or other surface contamination.

As genes related to growth and reproduction may be independent of genes related to the immune system, the routine mass-rearing process in artificial conditions could lead to a decrease of immune fitness. In shrimp aquaculture, where disease resistance is a crucial component of productivity and consistency, domestication and breeding programmes consider the immune effectors as markers of individual selection. The selection of individuals with the highest immune capacity is based on real-time RT-PCR or microarray analysis of mRNA of the main immune genes such as penaeidins, hemocyanin, phenoloxidase, superoxide dismutase, LPS-binding protein and β-glucan-binding protein. Such a strategy aims not only at avoiding the decrease of immune response but also at increasing the general non-specific resistance against various types of pathogens.

In mosquitoes, several types of viruses have been described which are related to the baculoviruses [42,43], iridoviruses [44-46], and parvoviruses particularly in *Anopheles *and *Aedes *spp. [47]. It must be considered that founder field-collected mosquito specimens may be infected - yet healthy - carriers of mosquito pathogens, some of which might be vertically transmitted. As in shrimp aquaculture, the founders could be analysed through e.g. nested-PCR based on highly conserved sequences in order to eliminate asymptomatic carriers. Routine analysis could also be performed in order to confirm that the production continues to be virus-free.

In *Anopheles *specifically, several intracellular pathogens have been described, such as *Rickettsia *[48] and *Spiroplasma *[49]. Recent information in shrimp aquaculture has shown that such pathogens are efficiently transmitted vertically and horizontally, and when these occur in high prevalence can result in mortality and severe losses of fecundity and vigour. Consequently, control of intracellular bacteria is a priority.

### Performance standards in other insect SIT programmes

Large size of individuals is a well-proven indicator of general nutritional status including energy reserves available for dispersion. Adult male size has also been correlated with successful mating in *Cochliomyia hominivorax*, being even more important in one study than strain type [50]. Evidence is inconclusive to support its prediction of mating success in mosquitoes. Large females of *An. gambiae *have been observed to be preferred mates [51], but it has not been demonstrated whether females preferentially mate larger males: observations for *An. gambiae *suggest male size is irrelevant [52]. Until the general observation that male size is relevant in Diptera is discounted for anophelines, production methods must consider that not merely consistent size, but a particular size itself may be a valuable goal.

For fruit flies standard quality control protocols have been established for mass-reared flies e.g. for pupal size and weight, emergence, flight ability, longevity, sex ratio, and mating propensity [19,53,54]. Comparison of results of the same tests from different mass-rearing facilities has allowed the establishment of standards for each of the parameters for several fruit fly species with, and without, irradiation. Though such quality control tests do not directly measure the competitiveness of the insect, they do provide standard indicators of the consistency of the production process. When linked to male releases that are suppressing populations, this is a valuable quality control measure.

#### Experimental approaches

• *Experimental field cages should be constructed at potential release sites in which field-collected mosquito mating behaviour occurs and tests of release material can be conducted. These facilities should be capable of measuring longevity and competition effects with varied numbers of wild males present*.

• *An experimental system must be developed in which the individual effects of larval density, tactile interactions, food density and type and waste accumulation can be incontrovertibly and individually tested*.

• *Rapid, extensive and inexpensive genotyping methods are needed to determine the effectiveness of many different colonisation and introgression methods*.

• *Laboratory systems for automation and control of production must be developed*.

• *Colonisation cages must be developed and their effects on founder population sizes compared. This characteristic reasonably reflects the probable long-term level of selection*.

• *Calculation methods should be developed that allow factory management of production characteristics (numbers, size, age etc.) to predict population control effects rather than merely numerical production outcomes*.

To measure competitiveness, a complementary set of data is required from tests in which sterile males compete sexually in a semi-natural environment with the feral population - the *mating performance field test *[55]. Sterility induced in open field tests can be obtained after sterile insects are released in semi- or isolated infested areas. Competitiveness is described by several indices: monitoring of the wild adult population, the level of sterility in wild females, or the level of sterility in eggs collected from dissected infested natural or artificial fruit in the field. Unlike anopheline mosquitoes, fruit flies respond to different types of attractants, which make mark-release-recapture tests more feasible to determine competitiveness, and its related components, dispersal and survival.

The history of experimental genetic control of mosquitoes is replete with stories of males that performed well in cage mating studies, but performed poorly in the field e.g. [56,57]. In contrast are examples in which laboratory competitiveness was consistent with adequate field performance [58]. As candidate release strains must necessarily pass laboratory tests before distribution, it serves as a useful quality control character whose use should be continued.

## Conclusion

Basic questions about the biology of mosquitoes related to colonisation and production are intellectually fascinating and immediately suggest numerous field and laboratory experiments that are also relevant to control efforts. Some of these, such as tests of diets and cage designs, will proceed rapidly and simply. Other difficult observations such as those of the genetics and significance of stenogamy will require much greater creativity. However, the achievements of past mosquito SIT efforts, using tools that might be considered crude by today's standards, provide a promising foundation on which to accomplish higher quality and better economics for colonisation and production.

## Competing interests

The authors declare that they have no competing interests.

## Authors' contributions

MQB coordinated the writing and wrote much of the discussion on colonisation, and production with PIH and HCB. BGJK contributed in the mating discussion, EM the disease overview, and CC the section on quality control. ASR critiqued and revised all sections. All authors read and approved the final manuscript.

## Supplementary Material

Additional file 1 **Status of colonisation of major anophelines**. Lists species, vector status according to the World Health Organisation, and status regarding whether it has been colonised and how mating was accomplished. Click here for file

## References

[B1] Baker RH (9645). Mating problems as related to the establishment and maintenance of laboratory colonies of mosquitoes. Bull World Health Organ.

[B2] Howell P, Knols BGJ (2009). Male mating biology. Malar J.

[B3] Fried M (1971). Determination of sterile-insect competitiveness. J Econ Entomol.

[B4] Menge DM, Guda T, Zhong DB, Pai A, Zhou GF, Beier JC, Gouagna L, Yan GY (2005). Fitness consequences of *Anopheles gambiae *population hybridization. Malar J.

[B5] Howell PI, Benedict MQ (2009). Mating competitiveness of *Anopheles arabiensis *males as a function of transgenic state and genetic similarity to females. J Insect Behav.

[B6] Norris DE, Shurtleff AC, Touré YT, Lanzaro GC (2001). Microsatellite DNA polymorphism and heterozygosity among field and laboratory populations of *Anopheles gambiae *s.s. (Diptera: Culicidae). J Med Entomol.

[B7] Munstermann LE (1994). Unexpected genetic consequences of colonization and inbreeding: allozyme tracking in *Culicidae *(Diptera). Ann Entomol Soc Am.

[B8] Matthews TC, Craig GB (1989). Isozyme polymorphisms maintained by lethal loci in inbred strains of *Aedes triseriatus*. J Hered.

[B9] Knop NF, Asman SM, Reisen WK, Milby MM (1987). Changes in the biology of *Culex tarsalis *(Diptera: Culicidae) associated with colonization under contrasting regimes. Environ Entomol.

[B10] Brooke BD, Hunt RH, Chandre F, Carnevale P, Coetzee M (2002). Stable chromosomal inversion polymorphisms and insecticide resistance in the malaria vector mosquito *Anopheles gambiae *(Diptera: Culicidae). J Med Entomol.

[B11] Seawright JA, Kaiser PE, Narang SK (1991). A unique chromosomal dimorphism in species-A and species-B of the *Anopheles quadrimaculatus *complex. J Hered.

[B12] Rutledge LC, Ward RA, Bickley WE (1970). Experimental hybridisation of geographic strains of *Anopheles stephensi *(Diptera: Culicidae). Ann Entomol Soc Am.

[B13] Marchand RP (1985). A new cage for observing mating behavior of wild *Anopheles gambiae *in the laboratory. J Am Mosq Control Assoc.

[B14] Fraccaro M, Tiepolo L, Laudani U, Marchi A, jayakar SD (1977). Y chromosome controls mating behaviour on *Anopheles *mosquitoes. Nature.

[B15] Kaiser PE, Bailey DL, Lowe RE, Seawright JA, Dame DA (1979). Mating competitiveness of chemosterilized males of a genetic sexing strain of *Anopheles albimanus *in laboratory and field tests. Mosq News.

[B16] Alphey L (2002). Re-engineering the sterile insect technique. Insect Biochem Mol Biol.

[B17] Papathanos PA, Bossin HC, Benedict MQ, Catteruccia F, Malcolm CA, Alphey L, Crisanti A (2009). Sex separation strategies: past experience and new approaches. Malar J.

[B18] Haeger JS, O'Meara GF (1970). Rapid incorporation of wild genotypes of *Culex nigripalpus *(Diptera: Culicidae) into laboratory-adapted strains. J Econ Entomol.

[B19] Enkerlin W, Bakri A, Caceres C, Cayol JP, Dyck A, Feldmann U, Franz G, Parker A, Robinson A, Vreysen M (2003). Insect pest intervention using the sterile insect technique: Current status on research and on operational programs in the world. Recent Trends on Sterile Insect Technique and Area-Wide Integrated Pest Management - Economic Feasibility, Control Projects, Farmer Organization and Bactrocera dorsalis Complex Control Study -".

[B20] Fisher K, Caceres C, Tan K-H (2000). A filter rearing system for mass reared genetic sexing strains of Mediterranean fruit fly (Diptera: Tephritidae). Area-wide Control of Fruit Flies and Other Insect Pests, Joint Proceedings of the International Conference on Area-wide Control of Insect Pests and of the Fifth International Symposium on Fruit Flies of Economic Importance, Penang, Malaysia, 1-5 June 1998.

[B21] Trembley HL (1944). Mosquito culture technique. Mosq News.

[B22] Dyck VA, Reyes Flores J, Vreysen MJB, Regidor Fernandez EE, Teruya T, Barnes B, Gomez Riera P, Lindquist D, Loosjes M, Dyck VA, Hendrichs J, Robinson AS (2005). Management of area-wide integrated pest management programmes that integrate the sterile insect technique. Sterile Insect Technique Principles and Practice in Area-Wide Integrated Pest Management.

[B23] Rao TR (1974). Research on genetic control of mosquitoes in India: Review of the work of the WHO/ICMR research unit, New Delhi. J Commun Dis.

[B24] Bailey DL, Lowe RE, Dame DA, Seawright JA (1980). Mass rearing the genetically altered MACHO strain of *Anopheles albimanus *Wiedemann. Am J Trop Med Hyg.

[B25] Feldmann AM, Brouwer W, Meeussen J, Voshaar O (1989). Rearing of larvae of *Anopheles stephensi*, using water replacement, purification and automated feeding. Entomol Exp Appl.

[B26] Dadd RH, Kleinjan JE (1976). Chemically defined dietary media for larvae of the mosquito *Culex pipiens *(Diptera: Culicidae): effects of colloid texturizers. J Med Entomol.

[B27] Dadd RH, Kleinjan JE, Sneller VP (1977). Development of several species of mosquito larvae in fully defined dietary media: preliminary evaluation. Mosq News.

[B28] Rosales-Ronquillo MC, Simons RW, Silverman PH (1973). Aseptic rearing of *Anopheles stephensi *(Diptera: Culicidae). Ann Entomol Soc Am.

[B29] Munderloh UG, Kurtti TJ, Maramorosch K (1982). *Anopheles stephensi *and *Toxorhynchites amboinensis*: aseptic rearing of mosquito larvae on cultured cells. J Parasitol.

[B30] Reisen WK, Milby MM, Asman SM, Bock ME, Meyer RP, McDonald PT, Reeves WC (1982). Attempted suppression of a semi-isolated *Culex tarsalis *population by the release of irradiated males: a second experiment using males from a recently colonized strain. Mosq News.

[B31] Dadd RH, Kleinjan JE, Asman SM (1988). Eicosapentaenoic acid in mosquito tissues: differences between wild and laboratory-reared adults. Environ Entomol.

[B32] Dadd RH, Kleinjan JE (1978). An essential nutrient for the mosquito *Culex pipiens *associated with certain animal derived phospholipids. Ann Entomol Soc Am.

[B33] Huho BJ, Ng'habi KR, Killeen GF, Nkwengulila G, Knols BGJ, Ferguson HM (2007). Nature beats nurture: a case study of the physiological fitness of free-living and laboratory-reared male *Anopheles gambiae s.l*. J Exp Biol.

[B34] Timmermann SE, Briegel H (1993). Water depth and larval density affect development and accumulation of reserves in laboratory populations of mosquitoes. Bull Soc Vector Ecol.

[B35] Roberts D (1998). Overcrowding of *Culex sitiens *(Diptera: Culicidae) larvae: population regulation by chemical factors or mechanical interference. J Med Entomol.

[B36] Richie JP, Mills BJ, Lang CA (1987). Correction of a glutathione deficiency in the aging mosquito increases its longevity. Proc Soc Exp Biol Med.

[B37] Richie JP, Mills BJ, Lang CA (1986). Dietary nordihydroguaiaretic acid increases the life span of the mosquito. Proc Soc Exp Biol Med.

[B38] Bangs MJ, Soelarto T, Barodji, Wicaksana BP, Boewono DT (2002). Colonization of *Anopheles maculatus *from Central Java, Indonesia. J Am Mosq Control Assoc.

[B39] Benedict MQ, Hood-Nowotny R, Howell PI, Wilkins EE (2008). Methylparaben in *Anopheles gambiae s.l*. sugar meals increases longevity and malaria oocyst abundance but is not a preferred diet. J Insect Physiol.

[B40] Feldmann U, Ochieng'-Odero JPR (1994). Some quality control parameters used in the rearing of tsetse flies. Techniques of insect rearing for the development of integrated pest and vector management strategies.

[B41] Feldmann U, Ochieng'-Odero JPR (1994). Guidelines for the rearing of tsetse flies using the membrane feeding technique. Techniques of insect rearing for the development of integrated pest and vector management strategies.

[B42] Hall DW, Fish DD (1974). A baculovirus from the mosquito *Wyeomyia smithii*. J Invertebr Pathol.

[B43] Becnel JJ, White SE, Moser BA, Fukuda T, Rotstein MJ, Undeen AH, Cockburn A (2001). Epizootiology and transmission of a newly discovered baculovirus from the mosquitoes *Culex nigripalpus *and *C. quinquefasciatus*. J Gen Virol.

[B44] Buchatsky LP (1977). An iridovirus from larvae of *Culiseta annulata *and *Culex territans*. Acta Virol.

[B45] Marina CF, Ibarra JE, Arredondo-Jimenez JI, Fernandez-Salas I, Liedo P, Williams T (2003). Adverse effects of covert iridovirus infection on the life history and demographic parameters of *Aedes aegypti*. Entomol Exp Appl.

[B46] Marina CF, Ibarra JE, Arredondo-Jimenez JI, Fernandez-Salas I, Valle J, Williams T (2003). Sublethal iridovirus disease of the mosquito *Aedes aegypti *is due to viral replication not cytotoxicity. Med Vet Entomol.

[B47] Rwegoshora RT, Baisley KJ, Kittayapong P (2000). Seasonal and spatial variation in natural densovirus infection in *Anopheles minimus s.l*. in Thailand. Southeast Asian J Trop Med Publ Health.

[B48] Venters D, Howells RE, Davies EE (1971). Rickettsia-like organisms in the mid-gut of *Anopheles stephensi*. Trans R Soc Trop Med Hyg.

[B49] Shaikh AA, Johnson JrWE, Stevens C, Tang AY (1987). The isolation of spiroplasmas from mosquitoes in Macon Country, Alabama. J Am Mosq Control Assoc.

[B50] Alley DA, Hightower BG (1966). Mating behavior of the screw-worm fly as affected by differences in strain and size. J Econ Entomol.

[B51] Okanda FM, Dao A, Njiru BN, Arija J, Akelo HA, Touré Y, Odulaja A, Beier JC, Githure JI, Yan G (2002). Behavioural determinants of gene flow in malaria vector populations: *Anopheles gambiae *males select large females as mates. Malar J.

[B52] Charlwood JD, Pinto J, Sousa CA, Ferreira C, Do Rosario VE (2002). Male size does not affect mating success (of *Anopheles gambiae *in Sao Tome). Med Vet Entomol.

[B53] Enkerlin W (2003). Economics of area-wide SIT control programs. Recent Trends on Sterile Insect Technique and Area-Wide Integrated Pest Management - Economic Feasibility, Control Projects, Farmer Organization and Bactrocera dorsalis Complex Control Study -".

[B54] FAO IAEA USDA (2003). Manual for product quality control and shipping procedures for sterile mass-reared tephritid fruit flies.

[B55] Cayol JP, Vilardi J, Rial E, Vera MT (1999). New indices and method to measure the sexual compatibility and mating performance of *Ceratitis capitata *(Diptera: Tephritidae) laboratory strains under field cage conditions. J Econ Entomol.

[B56] Davidson G, Odetoyinbo JA, Colussa B, Coz J (1970). Field attempt to assess the mating competitiveness of sterile males produced by crossing two member species of the *Anopheles gambiae *complex. Bull World Health Organ.

[B57] Baker RH, Reisen WK, Sakai RK, Hayes CG, Aslamkhan M, Saifuddin UT, Mahmood F, Perveen A, Javed S (1979). Field assessment of mating competitiveness of male *Culex tritaeniorhynchus *carrying a complex chromosomal aberration. Ann Entomol Soc Am.

[B58] Benedict MQ, Robinson AS (2003). The first releases of transgenic mosquitoes: an argument for the sterile insect technique. Trends Parasitol.

