# Supplementary material for: Colonisation and mass rearing: learning from others
Source: Malar J. 2009 Nov 16;8(Suppl 2):S4. doi: 10.1186/1475-2875-8-S2-S4 (PMC2777326; doi:10.1186/1475-2875-8-S2-S4)
Supplement: Additional file 1 — Status of colonisation of major anophelines. Lists species, vector status according to the World Health Organisation, and status regarding whether it has been colonised and how mating was accomplished. [file 1475-2875-8-S2-S4-S1.pdf]

Status of colonization of major anophelines.

**Species (<u>major vectors according to WHO underlined</u>)**	**Region**	**Mating Status**[1]
An. aconitus	South Pacific	colonized
<u>*An. albimanus*</u>	Americas	S
An. albitarsis	Americas	F/S
<u>*An. annularis*</u>	Indian subcontinent	F/S
An. anthropophagus	Asia	colonized
<u>*An. aquasalis*</u>	Americas	S
<u>*An. arabiensis*</u>	Africa	S
An. argyritarsis	Americas	no colony
<u>*An. atroparvus*</u>	Europe	S
An. aztecus	Americas	colonized
An. balabacensis ss	Southeast Asia	F
An. balabacensis sl	Southeast Asia	F
An. bancroftii	South Pacific	no colony
An. bellator	Americas	no colony
An. braziliensis	Americas	no colony
An. bwambae	Africa	S
An. campestris	Southeast Asia	colonized
An. claviger	Mediterranean	no colony
An. cruzii	Americas	no colony
<u>*An.. culicifacies*</u>	Indian subcontinent	F/S
An. daeneorum	Americas	F

An. darlingi	Americas	F
An. dirus	Southeast Asia	F
An. donaldi	South Pacific	no colony
An. dravidicus	Southeast Asia	no colony
An. elegans	Indian subcontinent	F
An. farauti	South Pacific	F/S
An. flavirostris	South Pacific	no colony
An. fluviatilis	Indian subcontinent	colonized
An. freeborni ss	Americas	S
An. funestus	Africa	S
An. gambiae ss	Africa	S
An. hackeri	Southeast Asia	no colony
An. hermsi	Americas	S
An. hilli	South Pacific	A
An. Hispaniola	Mediterranean	no colony
An. introlatus	Southeast Asia	F
An. jeyporiensis	Indian subcontinent	no colony
An. karwari	South Pacific	no colony
An. koliensis	South Pacific	F
An. labranchiae	Mediterranean	F
An. letifer	Southeast Asia	F
An. leucosphyrus ss	Southeast Asia	F
An. ludlowae	Southeast Asia	no colony
An. maculates	Southeast Asia	F/S

An. mangyanu	Southeast Asia	no colony
An. marajoara	Americas	no colony
An. melas	Africa	S
An. merus	Africa	S
An. messeae	Europe	no colony
An. minimus	Southeast Asia	F/S
An. moucheti	Africa	no colony
An. multicolor	Africa	S
An. nearpunctulatus	Southeast Asia	no colony
An. neivai	Americas	no colony
An. nemophilous	Southeast Asia	no colony
An. nigerrimus	South Pacific	F
An. nili	Africa	no colony
An. nivipes	Southeast Asia	F
An. notanandai	Southeast Asia	no colony
An. nuneztovari	Americas	no colony
An. occidentalis	Americas	F
An. pattoni	Asia	colonized
An. pharoensis	Africa	S
An. philippinensis	South Pacific	F
An. pseudowillmori	Southeast Asia	colonized
An. psuedopunctipennis	Americas	F/S
An. pujutensis	Southeast Asia	no colony
An. pulcherrimus	Asia	S

An. punctimacula	Americas	no colony
An. punctulatus ss	South Pacific	F
An. diluvialus	Americas	S
An. inundatus	Americas	S
An. maverlius	Americas	S
An. smaragdinus	Americas	S
An. quadriannulatus	Africa	S
An. quadrimaculatus ss	Americas	S
An. riparis	Southeast Asia	no colony
An. sacharovi	Mediterranean	F/S
An. sawadwongporni	Southeast Asia	F
An. sergentii	Africa	F
An. sinensis	Asia	F/S
An. stephensi	Indian subcontinent	S
An. subpictus	Indian subcontinent	S
An. sulawesi	Southeast Asia	no colony
An. sundiacus	Indian subcontinent	colonized
An. superpictus	Mediterranean	colonized
An. takasagoensis	Asia	colonized
An. tessellates	Indian subcontinent	colonized
An. triannulatus	Americas	no colony
An. varuna	Indian subcontinent	no colony
An. whartoni	Southeast Asia	no colony
An. willmori	Southeast Asia	colonized

[1] S-stenogamous, F-forced copulation, ‘colonized’ indicates a colony was established but no data regarding mating. All references are available upon request.
